# Danshao Shugan Granule therapy for non-alcoholic fatty liver disease

**DOI:** 10.1186/s12944-022-01689-9

**Published:** 2022-08-23

**Authors:** Hui Wang, Zhongju Xu, Qi Wang, Shi Shu

**Affiliations:** 1 Department of Traditional Chinese Medicine, Shanghai Punan Hospital, No. 279 Linyi Road, Pudong New District, Shanghai, 200125 China; 2 Department of Preventive Treatment of Disease, Shanghai GuangHua Hospital of Integrated Traditional Chinese and Western Medicine, No. 540 Xinhua Road, Changning District, Shanghai, 200052 China

**Keywords:** *Danshao Shugan* Granules (DSSG), NF-кB, Non-alcoholic fatty liver disease (NAFLD), Silibinin, Traditional Chinese medicine (TCM)

## Abstract

**Background:**

*Danshao Shugan* Granules (DSSG), a traditional Chinese medicine (TCM), is given to protect the liver. The objective is to evaluate the mechanisms of the effects of DSSG on non-alcoholic fatty liver disease (NAFLD).

**Methods:**

260 patients with NAFLD were randomly allocated to positive control drugs rosiglitazone (*n* = 30) and Silibinin (*n* = 50) as well as DSSG (*n* = 130) and combined DSSG/Silibinin (*n* = 50) groups, from which 90 patients in the DSSG group were further subdivided into 3 groups (*n* = 30, each) depending on the severity of symptoms. In total 33 Sprague–Dawley rats were assigned to normal (*n* = 10) or 45% high-fat diet (*n* = 23) groups, from which 9 rats served as negative controls, 10 as model controls and 10 were treated with DSSG.

**Results:**

DSSG medications had significantly highest effects on B-ultrasonography finding improvements, and reductions of total cholesterol, triglyceride, aspartate transaminase and γ-glutamyl transpeptidase in NAFLD patients. Silibinin application only led to significantly highest alanine transaminase reductions and rosiglitazone medication to significantly highest fasting plasma glucose reductions. In a murine in vivo NAFLD model glucose (GLU), total cholesterol (TC) triacylglycerol (TG) as well as glutamic pyruvic transaminase (GPT), glutamic oxaloacetic transaminase (GOT) and gamma-glutamyl transferase (GGT) serum concentrations were all significantly reduced (*P* < 0.001) and the expression of nuclear factor-κB (NF‑κB) was significantly decreased in DSSG treated compared to untreated NAFLD animals (*P* < 0.001). In addition, the DSSG treated rats exhibited increased superoxide dismutase activity and reduced malondialdehyde values.

**Conclusions:**

DSSG was effective for treating NAFLD patients, which could be attributed to increased activity of superoxide dismutase, a decrease of malondialdehyde as well as reduced NF‑*κ*B activity in a NAFLD rat model.

## Background

Non-alcoholic steatohepatitis (NASH) is an acquired metabolic disease that is closely related to insulin resistance. It is the pathological stage when fatty liver develops into liver fibrosis and is commonly diagnosed in individuals presenting with type 2 diabetes, metabolic syndrome, obesity and/or hyperlipidemia [1, 2]. In recent years, with improvements in living standards and changes in diet the incidence of NASH has been increasing year by year in China [3]. Moreover, with the increase of obesity in children, the age of NASH shows a trend of developing at a younger age [2]. The incidence of non-alcoholic fatty liver disease (NAFLD) worldwide is about 25% [4], and its prevalence in China has been estimated to affect more than 15% of the population [5].

At present, the theory of "Two Hits" is widely accepted [6]. Based on the first hit comprising steatosis, the secondary hit involves oxidative stress and lipid peroxidation, which increases the release of inflammatory cytokines and causes hepatocellular fatty degeneration and affect the liver function. This process in turn triggers inflammation, necrosis and even fibrosis of fatty liver cells. During the second hit, nuclear factor-*κ*B (NF-*κ*B) activation is a major component associated with concomitant inflammation and reactive oxygen mediation, since NF-*κ*B is a key transcription factor involved in liver inflammation, liver fibrosis and apoptosis [7–9].

The present clinical treatment of NAFLD mainly includes lipid-lowering and antioxidation drug therapy as well as liver protection and insulin sensitization therapies [10]. Obeticholic acid, which regulates the activity of nuclear factors, is considered to be a promising new treatment for NAFLD, but unfortunately its side effects include itching as well as elevated low density lipoprotein and decreased high density lipoprotein concentrations [11]. It is noteworthy that lipid-lowering drugs (statins) can reduce the levels of proinflammatory cytokines in the plasma of NAFLD patients [12], but there are still many challenges in using statins in clinical practice [13]. In an insulin sensitizing drug study, it was reported that in contrast to metformin, particularly in individuals without diabetes, glitazones showed significant histological and biochemical benefits in patients with NAFLD [14]. However, disadvantages of glitazones include sodium retention, weight gain, increased serum transaminase and insulin resistance after drug withdrawal, and the potential risk of developing cardiovascular disease. In addition, thiazolidinediones are under suspicion of increasing the incidence of bladder cancer [15].

After many years of clinical experience, the *Danshao Shugan* Granules (DSSG) (Patent number ZL1163720) were created in Shanghai Punan Hospital, and found its main function to be liver protection and reduction of fat deposition, but the mechanisms for effects on NAFLD were not clear. In this present study the clinical effects and adverse reactions of DSSG in reducing blood glucose and lipids and protecting liver functions index were investigated by comparing DSSG with western medicines. In order to explore the mechanisms of DSSG at the cell molecular level the actions of DSSG on NF-*к*B expression in a rat NAFLD were also evaluated. The aim was to provide a molecular finding base for the clinical use of DSSG for NAFLD patients.

## Methods

### Patients

The study protocols were approved by the ethics committee of Shanghai Punan Hospital (approval number: 2013–12-18) and all enrolled subjects provided signed informed consent forms. Two hundred sixty patients with NAFLD were enrolled in Shanghai Punan Hospital from January 2013 to May 2016. The Chinese guidelines for the management of NAFLD were used for patient diagnosis [16]. Exclusion criteria were: patients with alcoholic liver, viral, autoimmune or hereditary liver disease; organ failure; other serious diseases such as heart, liver, kidney, hematopoietic system, mental illness and cancer; pregnant and lactating women; had used drugs affecting lipid metabolism in the 4 weeks prior to the study; long-term use of other drugs and combination therapies that may affect efficacy and safety; incomplete information and inability to cooperate in completing the study.

Traditional Chinese medicine (TCM) criteria were applied according to the NASH diagnosis and treatment project of the key specialty collaboration group of The Eleventh Five-Year Plan of State Administration of TCM of the People’s Republic of China (PRC). Syndrome differentiation was as follows: (1) Liver-QI stagnation accompanied by spleen deficiency and phlegm stagnation; (2) Stagnation of phlegm, stasis of the blood and heat conversion of dampness stagnancy; (3) Damp depression, blood stasis and liver-YIN deficiency. TCM symptoms and sign scores were: (1) Hypochondriac pain; Implicit swelling pain, no impact on work, 2 points; relatively heavy swelling pain that was continuous, 4 points; swelling pain was severe and affects work, 6 points. (2) Fatigue: Limb slightly tired, does not affect work, 2 points; limb weakness, barely impinges on work, 4 points; the whole body is weak and patient unwilling to move at all, 6 points. (3) Abdominal distension: Abdominal distension after eating, relieved later, 2 points; postprandial abdominal distension with slow relief, 4 points; abdominal distension all day, 6 points. (4) Stools: Defecate rarefied or uncomfortable, 1–2 times a day, 2 points; a loose stool more than 3 times a day, 4 points; diarrhea immediately after eating, 6 points.

### Treatment procedures

**Fig. 1:**
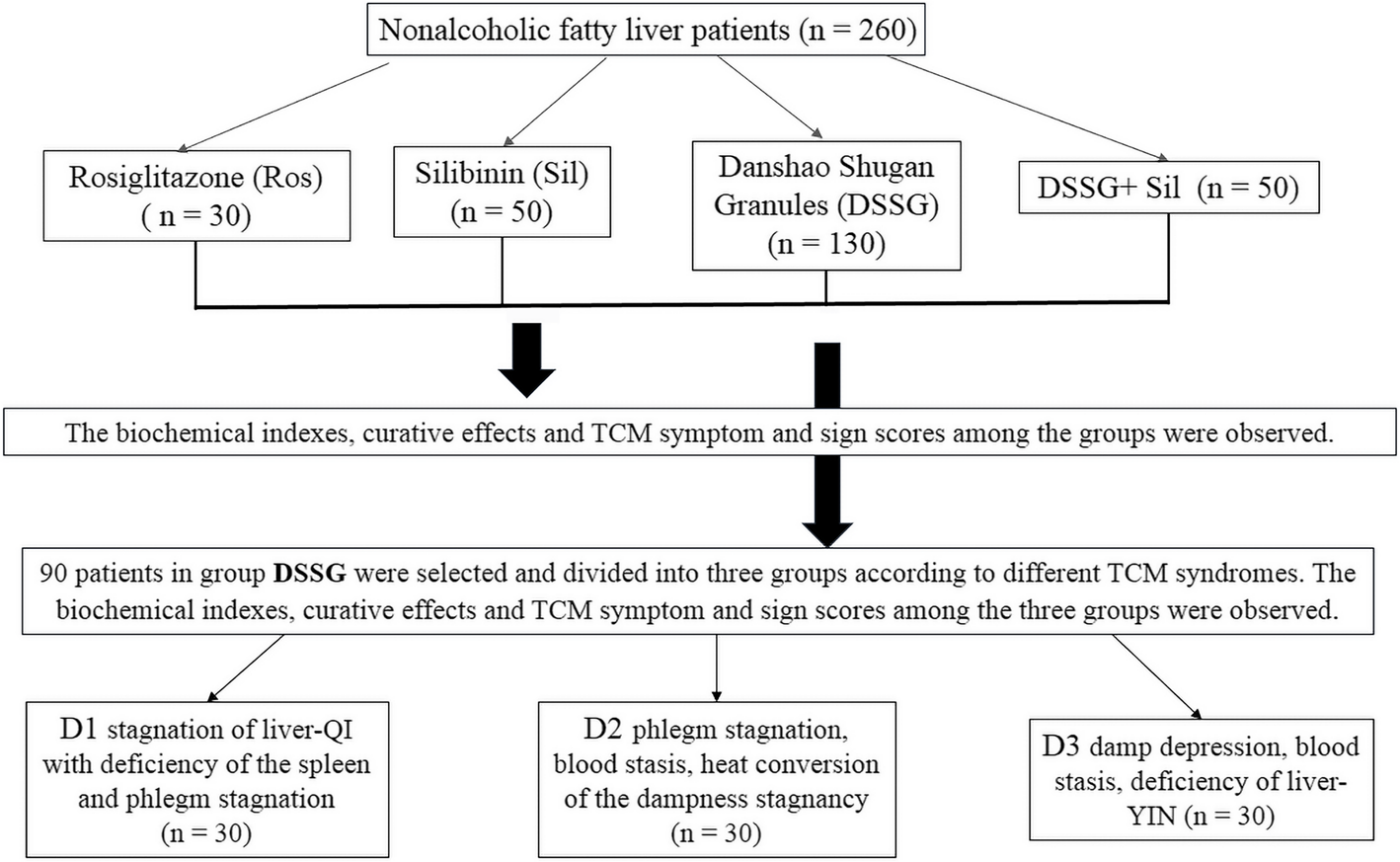
Flowchart of the study. NAFLD patients were treated with indicated drugs and outcomes were monitored for each regimen. Within the DSSG treated patients, 3 subgroups comprising 30 patients in each group were generated according to TCM specific categories and their intergroup outcomes have been compared

All NAFLD patients were given guidelines on requirements for diet control and appropriate exercise. The patients were randomly assigned to 4 groups: rosiglitazone, 30 patients (Ros); Silibinin, 50 patients (Sil); DSSG, 130 patients as well as 50 patients DSSG combined with Sil (DSSG + Sil). In total 90 patients in the DSSG group were screened and subdivided according to TCM classifications into a D1 group (liver-QI stagnation with spleen deficiency plus phlegm stagnation), a D2 group (phlegm stagnation, blood stasis, heat conversion of the dampness stagnancy) and a D3 group (damp depression, blood stasis, deficiency of liver-YIN), with 30 patients in each group (Fig. 1). The remaining 40 patients of the DSSG group did not belong to one of these classifications, but belonged to other TCM categories (such as liver depression with QI stagnation, etc.).

### Medications

#### Group Ros

Rosiglitazone hydrochloride (Yilixi, Zhejiang Wan Ma Pharmaceutical Co., Ltd. Manufacturing Approval Number: H20041408), 4 mg/time, once a day.

#### Group Sil

Silibinin capsules (Manufacturer: Tianjin TASLY Pharmaceutical Co, LTD, manufacturing approval number: 450707024); orally, 3 × 35 mg containing capsules, 3 times a day.

#### Group DSSG

Patients received DSSG (Composition: *Salvia miltiorrhiza* Bunge Lamiaceae 6 g, *Paeonia veitchii* Lynch Paeoniaceae 6 g and *Bupleurum komarovianum* Lincz. Apiaceae 5 g, *Curcuma aromatica* Sallsb. Zingiberaceae 6 g, *Cyperus rotundus* L. Cyperaceae 6 g, *Angelica sinensis* (Oliv.) Diels Apiaceae 5 g, *Fallopia japonica* Houtt. Polygonaceae 6 g, Radix Astragalus membranaceus (Fisch. ex Link) Bunge Fabaceae 8 g, *Artemisia capillaris* L. Asteraceae 9 g, *Isatis tinctoria* L. Brassicaceae 12 g, *Patrinia scabiosaefolia* Fisch Caprifoliaceae 12 g, *Artemisia argyi* H.Lév. and Vaniot Asteraceae 5 g, *Crataegus wilsonii* Sarg. Rosaceae 6 g, *Raphanus sativus* (L.) Domin Brassicaceae 6 g, *Cynanchum bungei* Decne. Apocynaceae 12 g, and *Schisandra chinensis* (Turcz.) Baill. Schisandraceae 5 g). The hospital entrusted Shanghai Yuan Pharmaceutical Co., Ltd to produce (Manufacturing Approval No: Z09130001), 12 g/packages, each containing the above dose proportions; 2 packages/time, were administered 3 times a day.

#### Group DSSG + Sil

DSSG (orally, 2 packages, 3 times a day) + Silibinin capsules (orally, 3 tablets, 3 times a day).

### Observation indices

#### Biochemical values

(1) Fasting serum lipids: total cholesterol (TC), triacylglycerol (TG). (2) Liver functions: alanine aminotransferase (ALT), aspartate transaminase (AST) and γ-glutamyl transpeptidase (GGT) concentrations; (3) Fasting plasma glucose (FPG). Biochemical indexes were carried out by the hospital laboratory and the results recorded before and after 16 weeks of treatment.

#### Imaging indexes

B-ultrasound examinations were performed before and after treatment for 16 weeks (the same technician used light, medium and heavy scans to evaluate the degree of fatty liver damage). The efficacy evaluation was based on clinical research guidelines for new TCMs to formulate the efficacy evaluation criteria for the severity of fatty liver using B-ultrasound. Efficacy was graded according to the following observations: *Clinical cure:* B-ultrasound reexamination found that the characteristics of NAFLD disappeared; *Obvious effect:* Grade of fatty liver B-ultrasound reduced by 2 levels; *Effective:* Grade of fatty liver B-ultrasound reduced by 1 level; *None-effective:* Failed to meet effective standards.

### *In vivo* rat experimental methods

Procedures involving laboratory rats were conducted following the ethical standards of Shanghai Punan Hospital and Chinese humane guidelines (Policy No. 2006 398). Specific-pathogen-free Sprague–Dawley rats (33 males, 8 weeks-old, weight range 220 ± 20 g) were supplied by Shanghai SLRC Laboratory Animal Co., Ltd. with Certificate No. SCXK (Shanghai) 2008–0016), Rats were housed and fed ad libitum for 5 days in a clean quiet environment (23 – 25 °C, relative humidity 50 – 70%).

**Fig. 2:**
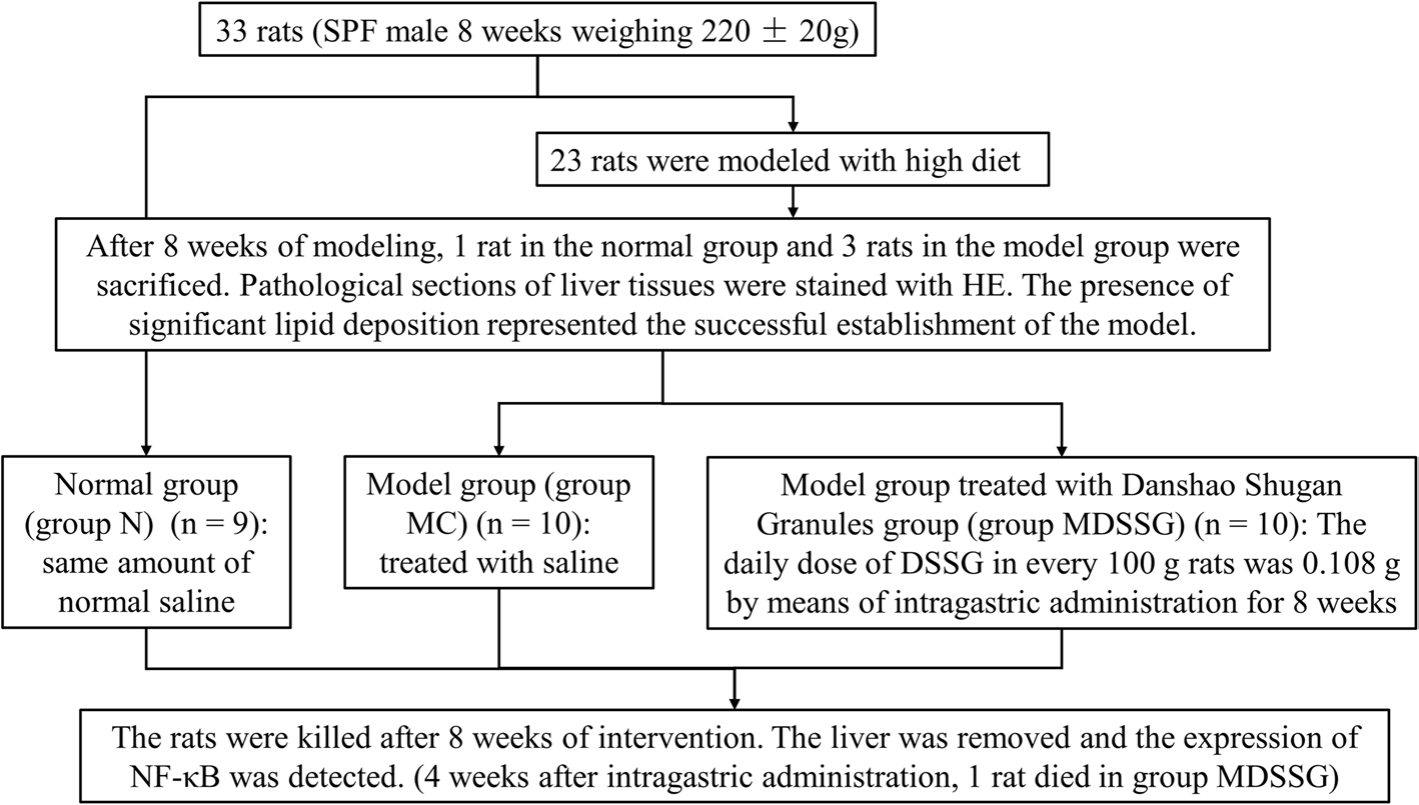
Flowchart of *in vitro* experiments. Rats in the NAFLD model group received high-fat diet for 8 weeks and were subsequently divided into a model group without treatment and a model DSSG group, which received DSSG for 8 weeks, with normal rats without special diet serving as controls

**Fig. 3:**
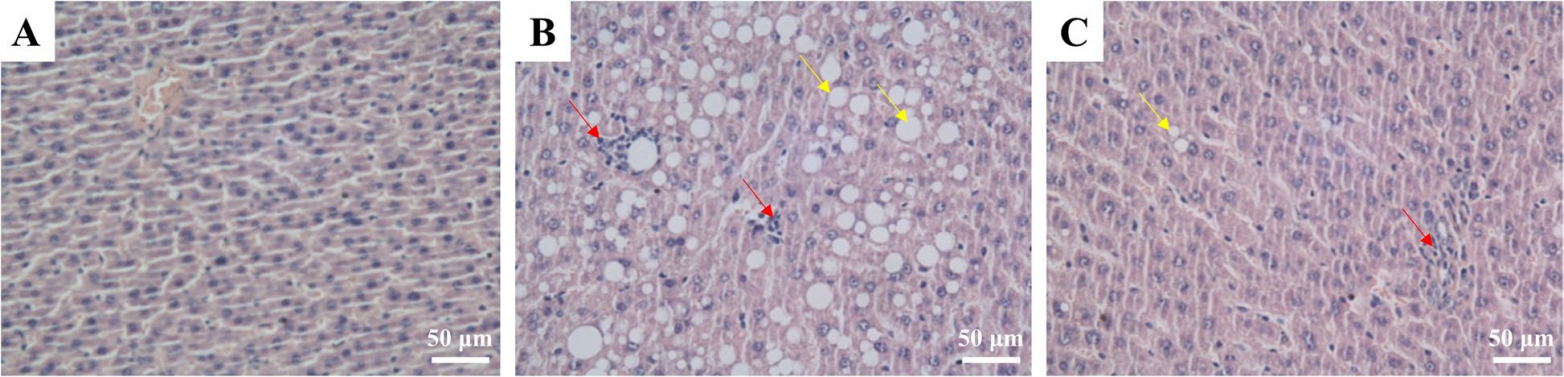
H&E staining images of rat liver tissues. **A** Control group, (**B**) NAFLD model group without treatment and (**C**) MDSSG group after application of DSSG. NAFLD model group liver hepatocytes without treatment included macrovesicular steatosis (yellow arrows) and inflammatory cell infiltrations (red arrows), which has been reduced by DSSG application. Magnification × 400

For the in vivo experiments, 33 rats were assigned to a control group with a normal diet (*n* = 10) and a model group fed a 45% high-fat diet (*n* = 23). The normal group was fed with purified feed with 10% fat content, while the NAFLD group was fed with purified feed with a 45% fat content, and all of the animals had access to drink water ad libitum. After successful model establishing, the remaining rats were subdivided into normal control (NC, *n* = 9), model control (MC, *n* = 10) and model DSSG (MDSSG, *n* = 10) groups, respectively. The NC and MC rats were given saline, whereas the DSSG group received DSSG. The dosage of DSSG in 200 g rats was 0.018 times than that for a normal weight person leading to DSSG doses of 0.108 g/100 g body weight, which was applied intragastrically (Fig. 2). Only male rats were chosen, since previous findings proposed male rats as more suitable for high-fat diet NAFLD models [17]. After 8 weeks, 1 rat in the control and 3 rats in the high-fat diet groups were sacrificed and liver tissue slices were examined and pathological sections of liver tissues stained with H&E. The presence of significant macrovesicular steatosis in the pathological sections represented the successful establishment of the model (Fig. 3A and B).

### Specimen collection and index determinations

The rats were humanely killed and weighed after 8 weeks of interventions. Rats were deeply anesthetized with chloral hydrate (0.3 mL/100 g body weight) and blood samples taken from the abdominal aorta to measure blood glucose and lipid concentrations, and to assess liver functions. After the rats were killed, the liver was immediately removed and washed in sterilized saline at 4 °C in an ice bath before being dried on filter paper. A small quantity of liver tissue was fixed in formalin for subsequent sectioning after paraffin-embedding. Liver homogenate was produced after immersing liver tissue (1 g) in normal saline (10 mL). The concentration of superoxide dismutase and malondialdehyde in liver tissues were measured, and expression of NF-κB by western blotting.

### Western blotting

Liver tissue (50 mg) was taken from the animals and l mL RIPA protein lysate was added. Next, 150 mM NaCl, 0.1% SDS, 0.5% sodium deoxycholate, 1% NP-40 and a complete protease inhibitor cocktail were added. The tissue was ground and homogenized in a ball mill. The resulting homogenate was centrifuged (4 °C, 16,000 rpm, 30 min) and the supernatant removed for analysis. A standard curve was constructed using Xylene brilliant cyanin G to determine the protein concentration of the liver tissue in each group. The samples were uniformly concentrated after determination of their protein concentrations. Subsequently, denaturation of 25 µg/mL of protein in loading buffer was carried out for 5 min at 100 °C.

After gel electrophoresis and transfer to PVDF membranes, blots were incubated with a primary antibody against NF-*κ*B (rabbit, Cell Signaling Technology, Inc. Danvers, MA, US) and GAPDH (mouse, Shanghai KangChen Bio-teck Co., Ltd.) at 4 °C overnight. After the application of appropriate secondary antibodies, electrochemiluminescence was employed to visualize the signals emitted by protein bands.

### Data analysis

SPSS Statistics for Windows (ver. 21.0) was employed for analysis of data. If the measured data were normally distributed, the mean ± SD was used to express the results. Single factor variance analysis was employed to make pairwise comparisons across multiple data groups. A *t*-test was used for normal distributions and variance homogeneity and a rank sum test for abnormal distribution. For enumeration data, a chi-squared, Wilcoxon rank sum or Mann Whitney U tests were used. *P* < 0.05 was deemed to be a significant finding.

## Results

### The changes of biochemical indicators during pre-and post-treatment

No significant differences between the 4 groups of patients regarding blood lipids, liver functions, other biochemical indexes, TCM symptoms, sign scores and B-ultrasonography scans before treatments were found (*P* > 0.05) (Table 1).

**Table 1:** Biochemical indicators of patients (blood lipids, liver function and blood sugar), B-ultrasound, TCM symptom and signs score before treatment (baseline) and after 16 weeks of treatment

		Ros	Sil	DSSG	*P-*value among three groups	DSSG + Sil	*P-*value (Sil *vs* DSSG + Sil)	*P-*value (DSSG *vs* DSSG + Sil)
Number		30	50	130		50		
TC (mmol/L)	Pre- treatment	6.55 ± 0.19	6.56 ± 0.28	6.46 ± 0.55	0.340	6.50 ± 0.46	> 0.05	> 0.05
	Post treatment	5.85 ± 0.15	6.41 ± 0.23	4.97 ± 0.33		6.02 ± 0.28		
	**Δ**	**-0.69 ± 0.29**	**-0.15 ± 0.29**	**-1.56 ± 0.95**	** < 0.05**	**-0.48 ± 0.41**	** > 0.05**	** < 0.05**
TG (mmol/L)	Pre- treatment	2.83 ± 0.13	2.80 ± 0.59	2.72 ± 0.90	0.695	3.03 ± 0.71	> 0.05	> 0.05
	Post treatment	2.26 ± 0.09	2.32 ± 0.51	1.80 ± 0.24		2.30 ± 0.41		
	**Δ**	**-0.57 ± 0.60**	**-0.48 ± 0.67**	**-0.99 ± 1.09**	** < 0.05**	**-0.73 ± 0.68**	** > 0.05**	** > 0.05**
ALT (U/L)	Pre- treatment	76.84 ± 3.51	75.41 ± 6.99	74.98 ± 16.39	0.792	76.47 ± 8.60	> 0.05	> 0.05
	Post treatment	71.18 ± 4.12	38.96 ± 4.51	44.01 ± 6.06		36.94 ± 4.01		
	**Δ**	**-5.66 ± 13.37**	**-36.45 ± 5.80**	**-32.78 ± 25.42**	** < 0.05**	**-39.53 ± 8.34**	** > 0.05**	** > 0.05**
AST (U/L)	Pre- treatment	72.42 ± 2.69	74.21 ± 8.27	73.08 ± 16.16	0.822	76.99 ± 12.02	> 0.05	> 0.05
	Post treatment	62.41 ± 2.32	54.20 ± 7.73	42.24 ± 4.96		53.26 ± 8.23		
	**Δ**	**-10.01 ± 17.60**	**-20.01 ± 8.84**	**-32.02 ± 19.73**	** < 0.05**	**-23.73 ± 8.31**	** > 0.05**	** < 0.05**
GGT (U/L)	Pre- treatment	71.02 ± 3.28	74.99 ± 9.08	76.40 ± 10.64	< 0.05	76.43 ± 9.95	> 0.05	> 0.05
	Post treatment	47.20 ± 2.54	64.26 ± 7.68	47.58 ± 6.21		60.09 ± 6.30		
	**Δ**	**-23.82 ± 13.97**	**-10.72 ± 10.79**	**-31.72 ± 18.89**	** < 0.05**	**-16.34 ± 8.31**	** > 0.05**	** > 0.05**
FPG (mmol/L)	Pre- treatment	6.09 ± 0.14	6.03 ± 0.29	5.89 ± 0.72	0.139	6.02 ± 0.36	> 0.05	> 0.05
	Post treatment	4.26 ± 0.08	5.76 ± 0.29	5.52 ± 0.58		5.63 ± 0.31		
	**Δ**	**-1.83 ± 1.19**	**-0.27 ± 0.28**	**-0.36 ± 0.72**	** < 0.05**	**-0.39 ± 0.36**	** > 0.05**	** > 0.05**
B-ultrasonography	Cure	4 (13.0%)	1 (2.0%)	40 (31.0%)	< 0.05	5 (10.0%)	> 0.05	< 0.05
	Obviously effective	12 (40.0%)	4 (8.0%)	32 (24.5%)	< 0.05	11 (22.0%)	< 0.05	> 0.05
	Effective	8 (27.0%)	24 (48.0%)	47 (36.0%)	0.139	23 (46.0%)	> 0.05	> 0.05
	Non-effective	6 (20.0%)	21 (42.0%)	11 (8.5%)	< 0.05	11 (22.0%)	< 0.05	< 0.05
	**Effective rate**	**24 (80.0%)**	**29 (58.0%)**	**119 (91.5%)**	** < 0.05**	**39 (78.0%)**	** < 0.05**	** < 0.05**
TCM symptom and signs score	Pre- treatment	14.00 ± 0.51	13.92 ± 2.39	13.72 ± 1.10	0.529	14.76 ± 2.60	> 0.05	< 0.05
	Post treatment	3.00 ± 0.31	9.10 ± 1.95	2.41 ± 0.43	< 0.05	5.04 ± 2.27	< 0.05	< 0.05
	**Δ**	**-11.00 ± 0.20**	**-4.82 ± 0.44**	**-11.31 ± 0.67**	** < 0.05**	**-9.72 ± 0.33**	** < 0.05**	** < 0.05**

Note: If the measured data were normally distributed, the mean ± standard deviation (SD) was used. Single factor variance analysis was used to make pairwise comparison among multiple groups. The test level was α = 0.05 and a *P*-value < 0.05 was considered to be statistically significant. **Δ**, value change before and after the rosiglitazone, Silibinin and DSSG treatments for the TCM symptoms and sign scores to compare the TCM syndrome. Group Ros: Rosiglitazone hydrochloride, 4 mg/time, once a day. Group Sil: Silibinin capsules, orally, 3 × 35 mg containing capsules, 3 times a day. Group DSSG: Patients received DSSG, 12 g/packages, each containing the above dose proportions; 2 packages/time, were administered 3 times a day. Group DSSG + Sil: DSSG (orally, 2 packages, 3 times a day) + Silibinin capsules (orally, 3 tablets, 3 times a day)

*ALT* alanine aminotransferase, *AST* aspartate amino transferase, *DSSG* Danshao Shugan Granules, *FPG* Fasting plasma glucose, *GGT* γ-glutamyl transpeptidase, *TC* total cholesterol, *TCM* traditional Chinese medicine, *TG* triacylglycerol

### DSSG lowers blood glucose and blood lipid levels and protects liver functions for NAFLD patients

By detecting changes in the biochemical indices of the 3 monotherapy Ros, Sil and DSSG groups after 16 weeks, it came out that the DSSG had the highest effects on TC and TG, AST and GGT reductions and rosiglitazone on FPG reduction. From all monotherapies, Silibinin could reduce ALT levels most efficiently (Table 1).

B-ultrasound examinations and the clinical cure of the DSSG group was most obvious with up to 31%, followed by the rosiglitazone (13%) and Silibinin (2%) groups, indicating that DSSG had the highest curative effect on NAFLD (Table 1). Therefore, the TCM syndrome change values (Δ) before and after the rosiglitazone application were compared by evaluations of TCM symptoms and sign scores. The TCM symptom and sign score changes in the DSSG and Ros groups were most obvious and higher than those in the Silibinin or Silibinin and DSSG groups (Table 1).

Next whether adding DSSG to Silibinin could promote an enhanced therapeutic effect than Silibinin alone was analyzed. As shown in Table 1, combination of Silibinin and DSSG improved outcomes of Silibinin monotherapy on B-ultrasound improvements and TCM scoring. However, the rates of TC and AST reductions under DSSG medication were significantly diminished by combination with Silibinin and the cure rate of 31% after DSSG monotherapy dropped to 10% after being combined with Silibinin medication.

In addition, the therapeutic effect of DSSG in patients having 3 different TCM syndromes were investigated and 90 selected patients of the DSSG group were divided into a D1 group (liver-QI stagnation with spleen deficiency and stagnation of phlegm), a D2 group (phlegm stagnation, blood stasis, heat conversion of the dampness stagnancy), and a D3 group (damp depression, blood stasis, deficiency of liver-YIN), with 30 patients in each group. No differences were detected between 3 groups with regard to age, gender, blood lipid concentrations, liver functions, other biochemical indexes, TCM symptoms and sign scores, and B ultrasonography scans (*P* > 0.05) before treatment (Table 2). Interestingly, there was a significant difference in TCM syndrome and sign scores between the 3 groups after treatment. The TCM syndrome and sign scores of patients with phlegm stagnation, stasis of the blood and heat conversion of the dampness stagnancy (D2) was the highest, followed by the syndrome of liver-QI stagnation with spleen deficiency and phlegm stagnation (D1); the lowest was the syndrome of damp depression, stasis of blood and liver-YIN deficiency (D3) (Table 2). The differences among the traditional Chinese syndrome scores compared to the included biochemical parameters might have been caused by changes of other biochemical factors, which were not investigated.

**Table 2:** Biochemical indicators (blood lipid, liver function and blood glucose), B-ultrasonography and TCM syndrome scores of three TCM syndromes types (before treatment and 16 weeks after DSSG treatment)

		D1	D2	D3	*P*-value
Number		30	30	30	
TC (mmol/L)	Pre- treatment	6.52 ± 0.19	6.50 ± 0.19	6.56 ± 0.17	0.439
	Post treatment	5.15 ± 0.12	4.61 ± 0.09	4.77 ± 0.11	
	**Δ**	**-1.37 ± 0.94**	**-1.89 ± 0.97**	**-1.79 ± 0.99**	**0.093**
TG (mmol/L)	Pre- treatment	2.77 ± 0.26	2.72 ± 0.22	2.76 ± 0.14	0.630
	Post treatment	1.77 ± 0.07	1.45 ± 0.06	1.66 ± 0.07	
	**Δ**	**-1.00 ± 1.13**	**-1.27 ± 1.17**	**-1.10 ± 0.69**	**0.586**
ALT (U/L)	Pre- treatment	75.58 ± 6.61	75.82 ± 5.16	77.92 ± 5.10	0.220
	Post treatment	43.01 ± 2.41	33.95 ± 0.87	45.32 ± 1.55	
	**Δ**	**-32.57 ± 28.86**	**-41.87 ± 25.43**	**-32.6 ± 26.26**	**0.308**
AST (U/L)	Pre- treatment	76.93 ± 2.36	76.60 ± 2.85	78.17 ± 3.52	0.100
	Post treatment	45.86 ± 1.46	41.04 ± 0.97	43.07 ± 1.44	
	**Δ**	**-31.07 ± 11.31**	**-35.56 ± 14.85**	**-35.1 ± 18.66**	**0.458**
GGT (U/L)	Pre- treatment	88.10 ± 3.75	86.20 ± 4.72	87.20 ± 3.37	0.190
	Post treatment	55.00 ± 1.08	48.51 ± 1.66	48.86 ± 1.87	
	**Δ**	**-33.10 ± 18.19**	**-37.69 ± 18.71**	**-38.34 ± 14.91**	**0.447**
FPG (mmol/L)	Pre- treatment	5.88 ± 0.18	5.95 ± 0.15	5.86 ± 0.12	0.060
	Post treatment	5.70 ± 0.13	5.55 ± 0.13	5.58 ± 0.10	
	**Δ**	**-0.18 ± 0.65**	**-0.40 ± 0.61**	**-0.28 ± 0.83**	**0.482**
B-ultrasonography	Cure	8 (26.7%)	10 (33.3%)	12 (40.0%)	0.549
	Obviously effective	11 (36.6%)	7 (23.4%)	6 (20.0%)	0.303
	Effective	9 (30.0%)	10 (33.3%)	10 (33.3%)	0.950
	Non-effective	2 (6.7%)	3 (10.0%)	2 (6.7%)	0.857
	**Effective rate**	**93.3%**	**90.0%**	**93.3%**	**0.857**
TCM symptom and signs score	Pre- treatment	14.07 ± 0.45	14.10 ± 0.44	14.20 ± 0.46	0.506
	Post treatment	2.33 ± 0.26 ^a^	2.13 ± 0.32 ^b^	2.86 ± 0.27 ^c^	< 0.001
	**Δ**	**-11.74 ± 0.19 **^**a**^	**-11.97 ± 0.12 **^**b**^	**-11.34 ± 0.19 **^**c**^	** < 0.001**

Note: If the measured data were normally distributed, the mean ± standard deviation (SD) was used. **S**ingle factor variance analysis was used to make pairwise comparison among multiple groups. ^a-c^ different lower case letters denotes a significant difference at *P* < 0.05 between the different treatment groups. The test level was α = 0.05 and a *P*-value < 0.05 was considered to be statistically significant. **Δ**, value change before and after the rosiglitazone, Silibinin and DSSG treatments for the TCM symptoms and sign scores to compare the TCM syndrome. D1 group, stagnation of liver-QI with deficiency of the spleen and phlegm stagnation; D2 group, phlegm stagnation, blood stasis, heat conversion of the dampness stagnancy; D3 group, damp depression, blood stasis, deficiency of liver-YIN

*ALT* alanine aminotransferase, *AST* aspartate amino transferase, *DSSG* Danshao Shugan Granules, *FPG* Fasting plasma glucose, *GGT* γ-glutamyl transpeptidase, *TC* total cholesterol, *TCM* traditional Chinese medicine *TG* triacylglycerol

### Establishment of the rat NAFLD model and detection of related indicators

In order to explore why DSSG can effectively treat patients with NAFLD, we successfully established a rat model of NAFLD. H&E staining of liver tissues revealed, that liver cells in the normal group were neatly arranged, without swelling, steatosis and inflammatory cell infiltration (Fig. 3A). In the model group, hepatic steatosis was obvious, accounting for more than 90% of the total field of vision. Some hepatocytes were enlarged and round with macrovesicular steatosis (red arrows) as well as inflammatory cell infiltration (blue arrow) (Fig. 3B). The MDSSG group had different degrees of steatosis, but it was significantly reduced compared with the model group, with reduced macrovesicular steatosis and no inflammatory cell infiltration (Fig. 3C).

We found that glucose, TC, TG, glutamic oxaloacetic transaminase, glutamic-pyruvic transaminase and GGT were increased in the model group, indicating the successful establishment of the NAFLD model in rats, an effect that could be reversed by administering DSSG solution. Compared with normal rats, the superoxide dismutase (SOD) activity decreased and malondialdehyde (MDA) content significantly increased in rat liver tissues of the model group. After the drug intervention, compared with the model group, the DSSG group exhibited increased SOD activity and reduced MDA content (*P* < 0.01, *P* < 0.01, Table 3).

**Table 3:** Comparison of biochemical indices in rats

	N (*n* = 8)^d^	MC (*n* = 10)	MDSSG (*n* = 9)^d^	*P*—value
GLU (mmol/L)	5.06 ± 0.36 ^c^	8.13 ± 0.32 ^a^	7.43 ± 0.22 ^b^	< 0.01
TC (mmol/L)	4.48 ± 0.65 ^c^	11.49 ± 0.45 ^a^	6.79 ± 0.53 ^b^	< 0.01
TG (mmol/L)	1.78 ± 0.26 ^c^	4.47 ± 0.49 ^a^	2.80 ± 0.36 ^b^	< 0.01
GOT (U/L)	39.12 ± 6.00 ^b^	77.40 ± 4.93 ^a^	42.67 ± 5.36 ^b^	< 0.01
GPT (U/L)	41.50 ± 4.52 ^b^	77.30 ± 4.92 ^a^	46.22 ± 4.54 ^b^	< 0.01
GGT (U/L)	64.63 ± 6.48 ^c^	115.30 ± 10.30 ^a^	75.00 ± 5.22 ^b^	< 0.01
SOD (U/mg prot)	417.39 ± 68.37 ^a^	214.87 ± 33.21 ^c^	295.11 ± 54.43 ^b^	< 0.01
MDA (nmol/mg prot)	2.61 ± 0.63 ^b^	3.97 ± 0.50 ^a^	3.16 ± 0.68 ^b^	< 0.01

Note: If the measured data were normally distributed, the mean ± standard deviation (SD) was used. For the animal experiments, the test data are expressed as the x ± SD, and single factor variance analysis was used to make pairwise comparison among multiple groups.The test level was α = 0.05 and a P-value < 0.05 was considered to be statistically significant.  ^a-c^ different lower case letters denote a significant difference at *P* < 0.05 between the different treatment groups.  ^d^ one rat in the MDSSG group and another one in the normal group have died and were not included into the measurements

*GGT* γ-glutamyl transpeptidase, *GLU* glucose, *GOT* glutamic oxaloacetic transaminase, *GPT* glutamic-pyruvic transaminase, *M*C model control group, *MDA* malondialdehyde, *MDSSG* Model group treated with DSSG, *N* normal group, *SOD* Superoxide dismutase, *TC* total cholesterol, *TCM* traditional Chinese medicine, *TG* triacylglycerol

**Fig. 4:**
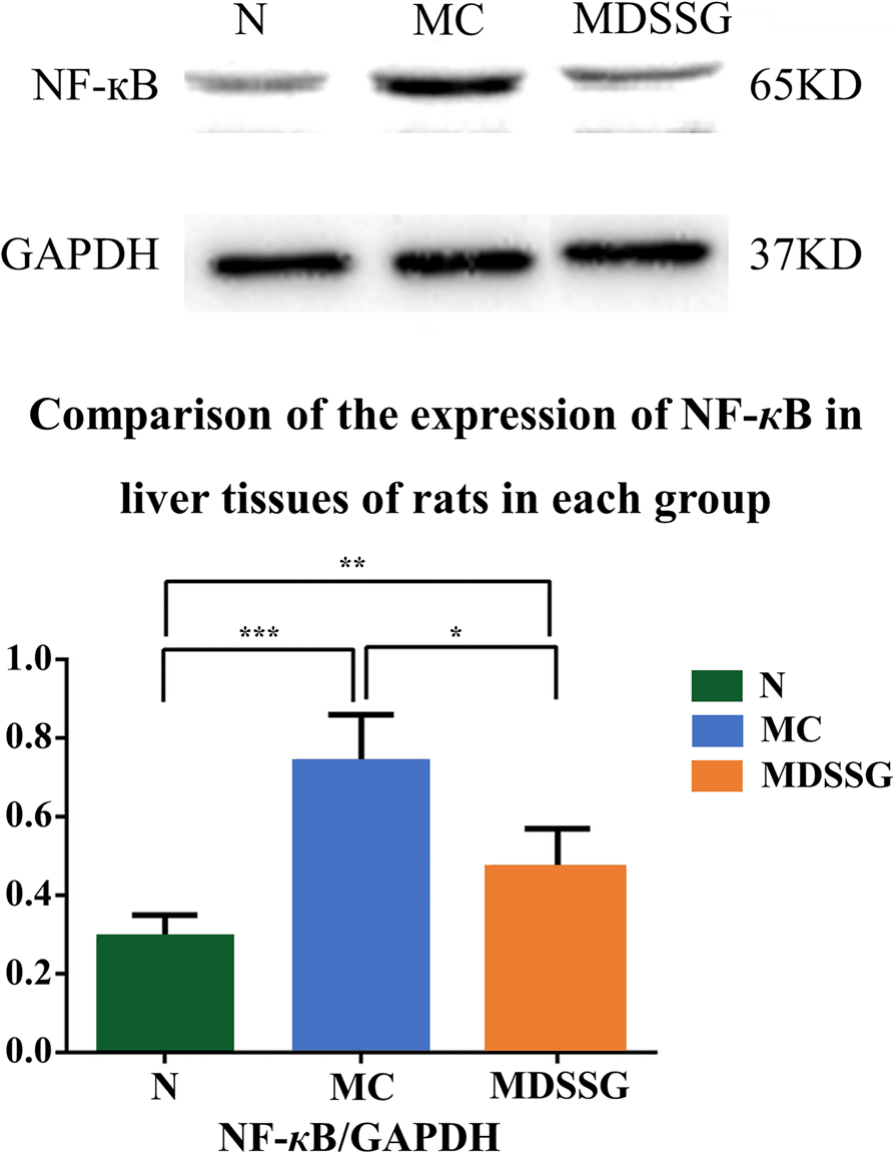
Comparison of the expression of NF-*к*B in liver tissues of normal control (N), NAFLD model control (MC) and NAFLD model DSSG treated rats (MDSSG)

Moreover, western blotting showed that the expression level of NF-*к*B was higher in the model group compared to the normal group (*P* < 0.001). After drug interventions, the levels of NF-*к*B in liver tissue of the DSSG group was decreased (*P* < 0.05). The results revealed that the levels of NF-*к*B in NAFLD rats of the DSSG group was decreased after treatment, suggesting that the significant therapeutic effect of DSSG might be related to lowering the expression levels of NF-*к*B (Fig. 4).

## Discussion

In the present study, NAFLD patients were randomly allocated to groups based on different drug treatment regimens to determine comprehensively the effects of reducing glucose and lipid levels and the protecting effects on liver function of DSSG. It was found that DSSG could reduce lipid profile and liver function parameters (TC, TG, ALT, AST, GGT and FPG levels). These effects may be related to the composition of DSSG, which is comprised of 16 Chinese herbs. Present pharmacological studies have proven that *Bupleurum komarovianum* can reduce serum transaminase levels, protect liver functions and inhibit liver damage [18]. *Salvia miltiorrhiza* reduces intracellular cholesterol levels and has anti-lipoprotein oxidation actions [19] as well improving the microcirculation of the liver, an increase blood flow, reduced triglyceride levels and promotion of the oxidation of fat, thereby reducing the fat content stored in the liver [18, 20]. *Paeonia veitchii* dilates blood vessels, improves the microcirculation and reduces the liver fibrosis process [21]. *Bupleurum komarovianum* can act on different fat metabolism pathways and has the effects of anti-fatty liver, anti-liver injury, liver benefit and enzyme and cholesterol lowering [22]. *Curcuma aromatic* has a strong inhibitory effect on the covalent binding of metabolites of CCl_4_ to liver microsomal lipids and proteins, thus protecting the integrity of liver cell membranes and has been shown to promote hepatocyte injury repair and protect hepatocytes [18]. The effective components of *Artemisia capillaris*, such as coumaric acid A, B, and 6, 7 dimethoxyoumarin, promote the secretion and excretion of bile. *Crataegus wilsonii* had an effect on the regulation of blood lipid levels and thus played a protective role limiting vascular damage caused by hyperlipidemia. The mechanism may be related to increased nitric oxide production and decreased endothelin synthesis [23]. The combination of drugs likely plays a role in ‘soothing’ the liver, eliminating stagnation, promoting blood circulation and dispersing stasis, dampness and turbidity. The present results show that DSSG can effectively improve clinical symptoms and liver functions, regulate blood lipid metabolism, and have a curative effect, but the exact mechanism of DSSG in treating NAFLD remains to be elucidated.

TCM treats NAFLD mainly based on the regulation of QI principle, promoting blood circulation and reducing phlegm, and has achieved a certain curative effect in the clinic. Mechanism research has been gradually deepened to the level of cell and molecular biology [24] and found that the SOD activity of rats with fatty livers caused by CCl_4_ combined with a high-fat and low-protein diet decreased significantly and reported that compound preparation of TCM could significantly inhibit the NF-*κ*B pathway in the liver and abdominal aorta of type 2 diabetic rats with NAFLD. Therefore, in the present study, the expression of SOD, MDA and NF-*κ*B were used as indicators to investigate the mechanism(s) of DSSG in preventing and treating NAFLD. The results of the in vivo studies indicated that the DSSG group had increased activity of SOD and a decrease in the content of MDA (*P* < 0.01, *P* < 0.01) and decreased expression of NF‑*κ*B compared to the model group, findings that were significant (*P* < 0.05). It is suggested that DSSG can inhibit lipid peroxidation and downregulate the expression of NF‑*κ*B in liver tissue of NAFLD rats, which may be one of the mechanisms underlying effective treatment of NAFLD. However, the chemical composition of DSSG is complex and its therapeutic effects may have the characteristics of multi-target and multi-channel actions.

Silibinin is an extract of *Silybum marianum* and is used in western medicine as medication for chronic liver disease with the reported common effects being antioxidant and a modulator of inflammation through inhibition of the NF-*κ*B pathway [25]. In addition, in several studies Silibinin has been shown to be an insulin sensitizer and could effectively reduce glucose plasma concentrations in type 2 diabetes mellitus and NAFLD patients, reflected in an enhanced homeostasis model assessment of insulin-resistance values [26–28]. However, addition of Silibinin to a DSSG medication partly reduced the effectivity of DSSG, which might be attributed to the detoxification properties of Silibinin [29, 30].

### Comparison to previous research: do the current findings add to existing knowledge

Taken together, DSSG is a novel plant derived TCM which is in contrast to Silibinin and other herbal drugs composed of a mixture of 16 herbs and thereby beneficial effects of otherwise also non-herbal single drug actions are combined, which reflected in superior outcomes after 16 weeks of NAFLD treatments compared to Silibinin and Rosiglitazone.

### Study strengths and limitations

Although its efficacy has been confirmed in clinical practice, this is the first time to evaluate the mechanisms of DSSG as a drug for treatment of NAFLD and its liver protection function in rat model. The shortcoming of the study was that it was not blinded and randomized and limited to the examination of NF-kB molecular expression in tissues of rats with NAFLD.

## Conclusions

The medication for NAFLD treatment includes lipid-lowering and insulin sensitizing drugs, but these agents have certain disadvantages such as sodium retention, weight gain, increased serum transaminase and insulin resistance after drug withdrawal. Since DSSG could improve B-ultrasonography findings and was effective in TC, triglyceride, AST and GGT reductions, it might serve as an alternative medication for the treatment of NAFLD, without producing the side effects of conservative lipid-lowering and insulin sensitizing drugs.

## Data Availability

The datasets used and/or analysed during the current study are available from the corresponding author on reasonable request.

## Abbreviations

ALT: Alanine aminotransferase
AST: Aspartate transaminase
DSSG: *Danshao Shugan* Granules
FPG: Fasting plasma glucose
GGT: γ-Glutamyl transpeptidase
GLU: Glucose
MC: Model control
MDA: Malondialdehyde
MDSSG: Model DSSG
NAFLD: Non-alcoholic fatty liver disease
NASH: Non-alcoholic steatohepatitis
NC: Normal control
SD: Standard deviation
SOD: Superoxide dismutase
TC: Total cholesterol
TCM: Traditional Chinese medicine
TG: Triacylglycerol
